# Development and Validation of an HPLC–PDA Method for Quality Control of Jwagwieum, an Herbal Medicine Prescription: Simultaneous Analysis of Nine Marker Compounds

**DOI:** 10.3390/ph18040481

**Published:** 2025-03-27

**Authors:** Chang-Seob Seo, Jeeyoun Jung, Sarah Shin

**Affiliations:** KM Science Research Division, Korea Institute of Oriental Medicine, Daejeon 34054, Republic of Korea; jjy0918@kiom.re.kr (J.J.); s.sarah@kiom.re.kr (S.S.)

**Keywords:** Jwagwieum, Joa-Gui Em, simultaneous analysis, quality control, HPLC–PDA

## Abstract

**Background/Objectives**: Jwagwieum (or Joa-Gui Em; JGE) consists of six herbal medicines, *Rehmannia glutinosa* (Gaertn.) DC., *Dioscorea japonica* Thunb., *Lycium chinense* Mill., *Cornus officinalis* Siebold & Zucc., *Poria cocos* Wolf, and *Glycyrrhiza uralensis* Fisch., and has been widely used to treat kidney-yin deficiency syndrome. In the present study, a high-performance liquid chromatography with photodiode array detector (HPLC–PDA) method for the simultaneous quantification of the nine components, i.e., gallic acid, 5-(hydroxymethyl)furfural, morroniside, loganin, liquiritin apioside, liquiritin, ononin, glycyrrhizin, and allantoin, was developed. **Methods**: The developed HPLC–PDA assay for quality control of JGE was validated with respect to linearity, limit of detection (LOD), limit of quantification (LOQ), recovery, and precision. **Results**: In the regression equation of the calibration curve, the coefficient of determination was ≥0.9980, and LOD and LOQ were 0.003–0.071 μg/mL and 0.010–0.216 μg/mL, respectively. Recovery and precision (relative standard deviation) were 96.36–106.95% and <1.20%, respectively. In this analytical method, nine compounds were detected at concentrations of 0.15–3.69 mg/lyophilized gram. **Conclusions**: The developed and validated analytical method could be used to obtain basic data for the quality control of JGE and related herbal prescriptions.

## 1. Introduction

Herbal medicine prescriptions, which mainly consist of two or more plant-derived herbal medicines, have been widely used in East Asian countries as traditional Korean medicine (TKM) in Korea, traditional Chinese medicine (TCM) in China, and Kampo medicine (KM) in Japan. They are known to be effective in improving various health problems, such as chronic diseases, aging, and immune regulation, through the combined effects of various medicinal herbs [1,2]. With the advancement of modern science and technology, research is actively being conducted to confirm the pharmacological effects and safety of herbal medicine prescriptions, such as TKM, TCM, and KM, thereby contributing to strengthening the scientific basis of herbal medicine and expanding its role in the global health system [3,4,5]. Here, quality control of TKM, TCM, and KM is also an important factor.

Jwagwieum (or Joa-Gui Em; JGE, Zuoguiyin in Chinese) is an herbal medicine prescription consisting of six herbal medicines: *Rehmannia glutinosa* (Gaertn.) DC. (Rehmanniae Radix Preparata), *Dioscorea japonica* Thunb. (Dioscoreae Rhizoma), *Lycium chinense* Mill. (Lycii Fructus), *Cornus officinalis* Siebold & Zucc. (Corni Fructus), *Poria cocos* Wolf (Poria Sclerotium), and *Glycyrrhiza uralensis* Fisch. (Glycyrrhizae Radix et Rhizoma Preparata cum Melle). JGE is representative prescription among TKM, TCM, and KM that has been widely used to treat kidney-yin deficiency syndrome [6,7].

Regarding the effect of JGE on kidney-yin deficiency syndrome, Han et al. [6] reported on the effect of improving renal damage and function in an animal model of unilateral ureteral obstruction, while Na et al. [7] reported on the renal protective effect through improvement of NLRP3 and TLR4/NF-κb signaling in an animal model of acute renal failure induced by ischemia/reperfusion.

Although some studies on the biological efficacy of JGE have been reported, there are few reports on quality control. Standardization studies are required to verify consistent biological efficacy and achieve quality control of herbal prescriptions, such as TKM, TCM, and KM. To develop an analytical method, the main components of the herbal medicines that constitute JGE were investigated: furan derivatives, 5-(hydroxymethyl)furfural, from *R. glutinosa* [8]; alkaloids, allantoin, from *D. japonica* [9]; alkaloids, betain, from *L. chinense* [10]; phenols, gallic acid, and iridoid glucosids, morroniside, sweroside, loganin, cornin, and cornuside, from *C. officinalis* [11,12]; triterpenoids, polyporenic acid C and pachymic acid, from *P. cocos* [13]; and flavonoids, liquiritin, liquiritigenin, liquiritin apioside, and ononin, and triterpenoid saponins, glycyrrhizin, from *G. uralensis* [14,15].

Today, the development of analytical methods for quality control of TKM, TCM, and KM is conducted using analytical techniques, such as high-performance liquid chromatography (HPLC) or ultra-performance liquid chromatography combined with photodiode array detection (PDA) and tandem mass spectrometry [16,17,18,19].

Herein, we developed and validated an HPLC–PDA method for simultaneous analysis, using nine marker compounds, to secure basic data for efficient quality control and clinical/nonclinical studies of JGE. The marker compounds were gallic acid (1), 5-(hydroxymethyl)furfural (2), morroniside (3), loganin (4), liquiritin apioside (5), liquiritin (6), ononin (7), glycyrrhizin (8), and allantoin (9) (Figure S1).

## 2. Results and Discussion

### 2.1. HPLC Profiling of Six Herbal Medicines Constituting JGE and Selection of Marker Compounds

The selection of the marker compounds was based on multiple criteria: (1) prior studies identifying the major bioactive components in the six medicinal herbs comprising JGE [8,9,10,11,12,13,14,15]; (2) the presence of these compounds in official herbal pharmacopoeias, including the Korean Pharmacopoeia (KP) and Chinese Pharmacopoeia (CP); and (3) their relevance as quality control markers in previous analytical studies. Notably, 5-(hydroxymethyl)furfural (*R. glutinosa*), glycyrrhizin (*G. uralensis*), loganin and morroniside (*C. officinalis*), and betain (*L. chinense*) are officially recognized in KP or CP as key quality markers. Furthermore, HPLC profiling was conducted to confirm the detectability and chromatographic separation of these compounds within JGE extracts. For this purpose, gradient elution of a distilled water (DW)–acetonitrile (ACN) solvent system, with added 0.1% (*v*/*v*) formic acid (FA), and a SunFire^TM^ C_18_ column (250 mm × 4.6 mm, 5 μm; Waters, Milford, MA, USA) were used for experiments. The major compounds of each raw herbal material investigated were as follows: 5-(hydroxymethyl)furfural from *R. glutinosa*; allantoin from *D. japonica*; betain from *L. chinensis*; morroniside, sweroside, loganin, methyl gallate, gallic acid, cornin, cornuside, and protocatechuic acid from *C. officinalis*; polyporenic acid C and pachymic acid from *P. cocos*; and liquiritin, liquiritin apioside, liquiritigenin, ononin, and glycyrrhizin from *G. uralensis*. The HPLC profiles for each of the considered herbal medicines and the major compounds are shown in Figure S2.

As shown in Figure S2, a total of 18 compounds were tested, as applicable to the JGE sample. Among them, allantoin and betain, the main components of *D. japonica* and *L. chinensis*, were not detected under the conditions of this analysis (Figure S2B,C). Betain was excluded from this analysis because of the lack of ultraviolet chromophores and short retention because of the amino-acid-based structure; allantoin was analyzed separately using a different column. Except for the two components allantoin and betain, 16 compounds were well separated and detected within 60 min without overlapping peaks (Figure S3A). However, only nine compounds (gallic acid, 5-(hydroxymethyl)furfural, morroniside, loganin, liquiritin apioside, liquiritin, ononin, glycyrrhizin, and allantoin) were detected in the JGE sample consisting of six herbal medicines (Figure S3B). Finally, nine compounds were selected as marker analytes for the quality control of JGE. Among the selected markers, allantoin was analyzed separately using a DW–ACN mobile phase system on a Luna NH_2_ column (250 mm × 4.6 mm, 5 μm, Phenomenex, Torrance, CA, USA).

### 2.2. Optimization of Operating Conditions for HPLC–PDA Simultaneous Analysis

To optimize the simultaneous analytical method for the eight analytes selected as marker compounds in JGE (Section 2.1), a previously reported analytical method was modified and applied [1]. The optimization was verified in three stages.

Stage I: In this stage, the resolution and peak shape of the markers were compared using several reversed-phase C_18_ columns, SunFire^TM^ (Waters), Kinetex (Phenomenex), Capcell Pak UG80 (Shiseido, Tokyo, Japan), and Hypersil GOLD (Thermo Fisher Scientific, San Jose, CA, USA). All of these columns had the same particle size of 5 μm, length 250 mm, and inner diameter 4.6 mm. As shown in Figure S4, excluding liquiritin apioside and liquiritin, the other markers had resolution > 1.5, which means they were fully separated, but liquiritin apioside and liquiritin showed differences in resolution depending on the column. Specifically, the resolution between the two compounds was 2.26, 1.14, 1.84, and 0.99 on the SunFire^TM^, Kinetex, Capcell Pak UG80, and Hypersil GOLD columns, respectively. In particular, gallic acid eluted with a broad peak shape on the Kinetex compared with the other columns (Figure S4B). In addition, tailing of gallic acid and 5-(hydroxymethyl)furfural occurred on the Hypersil GOLD column (Figure S4D). Because the resolution of the two compounds liquiritin apioside and liquiritin, were the best on the SunFire^TM^ column, this column was ultimately selected for further use.

Stage II: Following column selection, the peak shape and resolution were compared according to the acids added to the mobile phase: 0.1% (*v*/*v*) FA, 0.1% (*v*/*v*) trifluoroacetic acid (TFA), 0.1% (*v*/*v*) phosphoric acid (PA), and 1.0% (*v*/*v*) acetic acid (AA). As shown in Figure S5, the resolutions for all marker compounds ranged from 2.06 to 2.28 across tested conditions, indicating clear baseline separation. Since all values exceed the chromatographic threshold (resolution > 1.5), no significant variation in resolution was observed between conditions. Therefore, FA, which is frequently added to the mobile phase in HPLC or liquid chromatography–tandem mass spectrometry analysis, was selected.

State III: After determining the preferred type of column and the acid to be added to the mobile phase, the peak patterns of marker compounds according to the column temperature (30, 40, and 50 °C) were compared and evaluated (Figure S6). In the comparison of the resolution using the markers, all tested well at >1.5. However, in the case of the JGE sample, overlapping of gallic acid with a neighboring unknown peak at 30 °C was detected (Figure S6A-Sample solution). Furthermore, at 50 °C, the tailing phenomenon of gallic acid and 5-(hydroxymethyl)furfural (Figure S6C-Standard solution) and the overlap with the unknown peak of loganin in the JGE sample (Figure S6C-Sample solution) were observed. The optimal column oven temperature was subsequently determined to be 40 °C.

**Figure 1:**
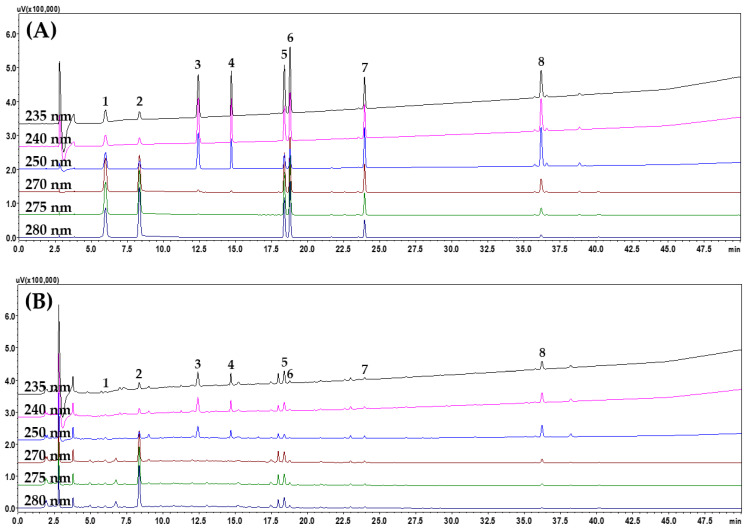
High-performance liquid chromatography with photodiode array detection (HPLC–PDA) chromatograms of a mixed standard (**A**) and Jwagwieum (JGE) sample (**B**) solutions. Gallic acid (1), 5-(hydroxymethyl)furfural (2), morroniside (3), loganin (4), liquiritin apioside (5), liquiritin (6), ononin (7), and glycyrrhizin (8). The concentration of each component in the measured standard solution is 10.00 μg/mL for 5-(hydroxymethyl)furfural, morroniside, and loganin; 20.00 μg/mL for gallic acid, liquiritin apioside, liquiritin, and ononin; and 100.00 μg/mL for glycyrrhizin.

Based on the above test results, the optimal analytical method for the simultaneous analysis of eight marker compounds in JGE samples was established; it included the following: a SunFire^TM^ C_18_ column, mobile phase of DW–ACN containing 0.1% (*v*/*v*) FA, and a column oven temperature of 40 °C. More detailed HPLC operating conditions are presented in Table S1. Representative HPLC chromatograms of a standard solution and JGE solution are presented in Figure 1.

**Figure 2:**
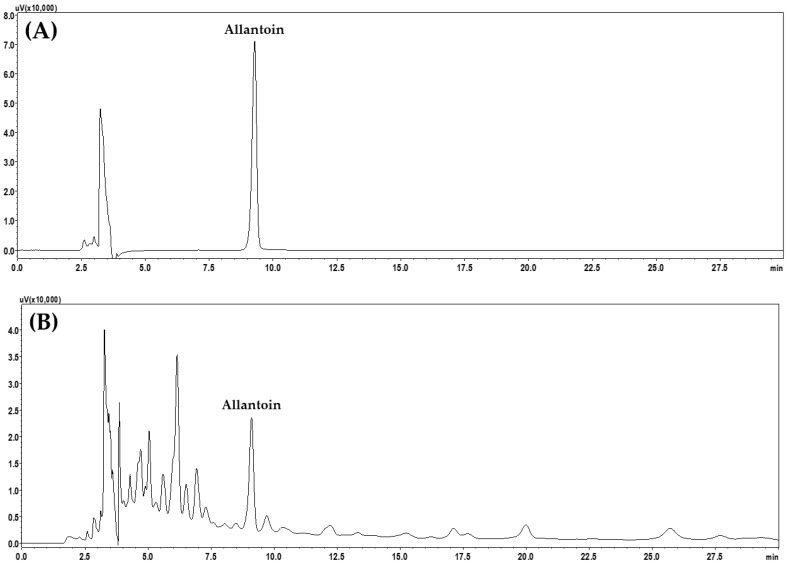
Chromatograms of allantoin standard compound (**A**) and JGE sample (**B**) recorded by HPLC–PDA at 210 nm.

Analysis of allantoin was performed using a previously reported analytical protocol [9]. Briefly, the experiment was conducted using a Luna NH_2_ column (250 mm × 4.6 mm, 5 μm, Phenomenex) maintained at 40 °C and an isocratic flow of DW–ACN mobile phase (Table S1). An HPLC chromatogram is shown in Figure 2.

Under the established analytical method, the performance of an HPLC device was measured based on the relative standard deviation (RSD, %) values of retention time and peak area using marker compounds. As shown in Tables S2 and S3, an excellent HPLC device performance was demonstrated: RSD ≤ 0.53%.

### 2.3. Method Validation of the Developed HPLC–PDA Assay

In this study, we validated the HPLC–PDA analytical method developed for quality control of JGE (herbal medicine prescription) by evaluating its linearity, sensitivity (limit of detection [LOD] and limit of quantification [LOQ]), accuracy (recovery), and precision using nine selected marker compounds. The validation data are tabulated in detail in Table 1, Table 2 and Table 3. The coefficient of determination (*r*^2^) used as a linearity validation factor was evaluated from the regression equation of the calibration curve tested in the concentration ranges of 0.16–10.00 μg/mL (gallic acid, morroniside, loganin, liquiritin, and ononin), 0.31–20.00 μg/mL (5-(hydroxymethyl)furfural), 0.47–30.00 μg/mL (liquiritin apioside), and 1.566–100.00 μg/mL (glycyrrhizin). The *r*^2^ values of the eight marker compounds in JGE samples were ≥0.9980, showing excellent linearity (Table 1). In addition, the LOD and LOQ concentrations, measured for sensitivity, were calculated to be 0.003–0.764 μg/mL and 0.010–2.315 μg/mL, respectively (Table 1). The recovery for accuracy evaluation was 96.36–106.95% (RSD ≤ 2.50%), showing excellent accuracy (Table 2). Precision (intraday and interday) was assessed by RSD (Table 3). For all marker compounds, the RSD values for precision were <1.20%. These results suggest that the HPLC–PDA assay developed in this analysis method is suitable as an analysis method for the quality assessment of JGE.

### 2.4. Stability Test

The stability of the marker compounds selected for the quality assessment of JGE in standard and sample solutions was measured to be 97.14–105.24% with RSD values ≤ 2.06% (Tables S4 and S5). This means that the stability of the nine marker compounds is guaranteed for at least 72 h, demonstrating the robustness of the analytical method for quality control applications. In addition, the marker compounds were stored in amber vials to prevent light-induced degradation. The stability data will further support the applicability of the method to routine quality control by ensuring consistency between batches of JGE extracts.

### 2.5. System Suitability Evaluation

System suitability for peak performance evaluation was assessed according to established criteria. Specifically, all parameters met the required thresholds: capacity factor (*k*) ≥ 1.16, selectivity factor (*α*) ≥ 1.03, resolution (*Rs*) ≥ 2.25, number of theoretical plates (*N*) ≥ 16,566.18, and symmetry factor (*S*) ≤ 1.19 (Table S6). The results confirmed that development of the analytical method was successfully conducted.

### 2.6. Simultaneous Quantitative Analysis of Nine Marker Compounds in JGE Samples

The analytical method, successfully optimized for the efficient quality control of JGE (herbal medicine prescription), was applied to real samples. Quantification was performed by monitoring the ultraviolet maximum absorption wavelength of each target compound: allantoin (210 nm), loganin (235 nm), morroniside (240 nm), ononin and glycyrrhizin (250 nm), gallic acid (270 nm), liquiritin apioside and liquiritin (275 nm), and 5-(hydroxymethyl)furfural (280 nm). In the freeze-dried JGE samples, nine marker compounds were quantified in amounts ranging from 0.15 to 3.69 mg/g (Table 4). Among these, glycyrrhizin, the primary marker of *G. uralenssis*, was the most abundant amounts ranged from 3.67 to 3.69 mg/g.

## 3. Materials and Methods

### 3.1. Plant Material

Among the six herbal medicines that constitute JGE, *R. glutinosa* was purchased from Shin Hung (Yeosu, Republic of Korea); *D. japonica*, *L. chinensis*, *C. officinalis*, and *P. cocos* were purchased from Sunilmulsan (Seoul, Republic of Korea); and *G. uralensis* was purchased from CK Pharm Co., Ltd. (Seoul, Republic of Korea) in July 2023. These companies are all specialized herbal medicine manufacturers that have received good manufacturing practice certification from The Korean Ministry of Food and Drug Safety. Further detailed information on these raw herbal medicines is given in Table S7. Each raw herb was morphologically identified by Dr. Goya Choi, Korea Institute of Oriental Medicine (KIOM, Naju, Republic of Korea). A specimen (2022HA01–1 to 2022HA01–6) of each raw herb has been deposited at the KM Science Research Division, KIOM.

### 3.2. Chemicals and Reagents

Nine reference standards (purity ≥ 98.0%) for the quality control of JGE were purchased from natural product manufacturers. The reference standards were sourced from Merck KGaA (Darmstadt, Germany), Wuhan ChemFaces Biochemical Co., Ltd. (Wuhan, China), Biopurify Phytochemicals Ltd. (Chengdu, China), and Shanghai Sunny Biotech Co., Ltd. (Shanghai, China). Detailed information on each reference standard is given in Table S8. Methanol (MeOH), ACN, and DW (all HPLC grade) were sourced from JT Baker (Phillipsburg, NJ, USA). ACS-grade FA (≥99.7%, catalog No. 33015, lot No. STBL0518) was purchased from Merck KGaA. HPLC-grade AA (≥99.7%, catalog No. A35, lot No. 193296) was obtained from Thermo Fisher Scientific (Waltham, MA, USA). Furthermore, HPLC-grade TFA (≥99.0%, catalog No. 302031, lot No. STBH6629) and PA (85%, catalog No. 49685, lot No. BCCF1913) were purchased from Merck KGaA.

### 3.3. Preparation of JGE Water Extract Sample

JGE water extract was manufactured by Purichems Co., Ltd. (Uiwang, Republic of Korea), a company specializing in natural product extract manufacturing. Briefly, according to the weights listed in Table S7, 31.5 L of water was added to six herbal medicines (total 3.15 kg), pressure extracted using electric extractor (Seoul, Republic of Korea) at 95 °C for 3 h, and then filtered using a filter cloth (10 μm). The filtrate was concentrated by vacuum decompression at 80 °C with a vacuum of −0.08 MPa and then freeze-dried using a freeze dryer (LP20, IlShinBioBase, Yangju, Republic of Korea). This yielded 827.5 g (yield 26.3%) of powder sample, which was then stored in a refrigerator (approximately 4 °C and 30% humidity) until use.

### 3.4. Preparation of Standard and Sample Solutions for the Simultaneous Quantification by the HPLC–PDA Assay

Stock solutions of the eight reference standard compounds used in this analytical assay were prepared in MeOH, concentration 1.0 mg/mL. The prepared solutions were stored (3 days) in a refrigerator at approximately 4 °C (30% humidity) and serially diluted prior to use. In the developed HPLC–PDA assay, a test solution for the simultaneous quantification of eight analytes, selected as marker compounds of JGE, was prepared by adding 10 mL of 70% MeOH to 100 mg of freeze-dried JGE sample, followed by ultrasonic extraction for 60 min. All solutions were filtered through a 0.2-μm membrane filter (GVS ABLUO, Sandford, ME, USA) prior to HPLC analysis.

### 3.5. Instrumental and Operating Conditions for Simultaneous HPLC–PDA Analysis

HPLC operating conditions for simultaneous determinations were applied based on a previously reported protocol [1]. Briefly, the method development was conducted using a Shimadzu Prominence LC-20A series HPLC system (Tokyo, Japan) controlled by LabSolution software (version 5.117, Tokyo, Japan). The chromatographic separation of the eight compounds (gallic acid, 5-(hydroxymethyl)furfural, morroniside, loganin, liquiritin apioside, liquiritin, ononin, and glycyrrhizin) was performed using the SunFire^TM^ reversed-phase column (250 mm × 4.6 mm, 5 μm, Waters), and allantoin was analyzed separately using a Luna NH_2_ column (250 mm × 4.6 mm, 5 μm, Phenomenex). The detailed HPLC operating conditions are presented in Table S1.

### 3.6. Analytical Method Validation of the Developed HPLC–PDA Assay

Validation of the HPLC–PDA analytical assay developed for the quality control of JGE was demonstrated by evaluating linearity, LOD, LOQ, recovery, and precision based on established guidelines [20]. Briefly, the regression equation for the calibration curve of each marker is expressed in the form *y* = a*x* + b, where *y* represents the peak area and *x* represents the concentration (μg/mL) of the reference standard. Linearity was assessed by evaluating the *r*^2^ value of the regression equation measured over different concentration levels. To assess sensitivity, the LOD and LOQ concentrations were calculated using the standard deviation of each *y*-intercept and the average slope obtained from the regression analysis of calibration curve data measured in triplicate. The calculations were performed using the following equations: LOD = 3.3 × *σ*/*S* and LOQ = 10 × *σ*/*S*, where *σ* represents the standard deviation of the *y*-intercept and *S* is the average slope of the regression equation. Recovery was used as an indicator for evaluating accuracy and measured five times (n = 5) using the standard addition method, which involved adding three different concentration levels (low, medium, and high) to a known sample. Recovery was calculated using the following formula: Recovery (%) = (detected amount − original amount)/spiked amount × 100. Finally, precision was assessed based on the RSD values of intraday (1 day, n = 5) and interday (3 day, n = 15) measurements.

### 3.7. Stability Test

The stability of each compound in MeOH solution was tested over 3 days (at 0, 6, 12, 24, 36, 48, and 72 h) using a standard solution containing mixed marker compounds. The concentration of each component in the tested standard solution was 10.00 μg/mL for 5-(hydroxymethyl)furfural, morroniside, and loganin; 20.00 μg/mL for gallic acid, liquiritin apioside, liquiritin, and ononin; and 100.00 μg/mL for glycyrrhizin. The initial measurement data were set to 100.0% and compared. In the case of allantoin (100.00 μg/mL in MeOH), since it was not detected on the C_18_ column, the test was conducted separately using a NH_2_ column suitable for the detection of allantoin. In addition, the stability of sample solutions prepared using 70% MeOH was tested for 3 days (at 0, 6, 12, 24, 36, 48, and 72 h). Allantoin was tested using a NH_2_ column in the same manner.

### 3.8. System Suitability Test

Parameters for evaluating peak performance in the developed HPLC–PDA analysis method—*k*, *α*, *Rs*, *N*, and *S*—were tested based on published guidelines [20,21]. The criteria for these parameters were set as follows: *k* > 1, *α* > 1, *Rs* ≥ 1.5, *N* > 2000, and *S* ≤ 2.0, and the peak performance of the developed analysis method was validated.

## 4. Conclusions

In this study, we developed, what we believe for the first time, an analytical method for the quality control of JGE, used in the treatment of kidney-yin deficiency syndrome in TKM, in which an HPLC–PDA system was used. The latter is a widely utilized technique for the development of analytical methods. The developed analytical method was demonstrated to be appropriate based on the evaluation of several parameters, including linearity (*r*^2^ > 0.9980), sensitivity (LOD: 0.003–0.071 μg/mL and LOQ: 0.010–0.216 μg/mL), accuracy (96.36–106.95%), precision (RSD < 1.20%), and system suitability. These findings confirm that the developed analytical method is highly reliable and suitable for the quality control of JGE and similar herbal prescriptions.

The significance of this study lies in its contribution to the standardization and regulatory assessment of herbal medicines. By establishing a validated analytical method, this study provides essential data that can be applied in quality assurance programs, ensuring consistency and efficacy of herbal medicine prescriptions.

Future research should explore the pharmacokinetics and bioavailability of these marker compounds in biological systems, enabling a better understanding of their therapeutic efficacy. Additionally, the analytical approach could be expanded to cover other traditional herbal prescriptions, further supporting the scientific standardization of TCMs, TKMs, and KMs.

## Figures and Tables

**Table 1:** Various parameters for each marker compound in a simultaneous HPLC analysis (n = 3).

Analyte ^a^	Detection Wavelength (nm)	Linear Range (μg/mL)	Regression Equation	*r* ^2 c^	LOD ^d^ (μg/mL)	LOQ ^e^ (μg/mL)	Retention Time (min)
1	270	0.16–10.00	*y* = 53,334.63*x* + 144.75	1.0000	0.004	0.011	5.99
2	280	0.31–20.00	*y* = 144,135.94*x* − 18,031.79	0.9997	0.041	0.125	8.34
3	240	0.16–10.00	*y* = 92,740.24*x* + 2003.38	1.0000	0.004	0.013	12.42
4	235	0.16–10.00	*y* = 56,300.13*x* + 1261.95	1.0000	0.006	0.020	14.71
5	275	0.47–30.00	*y* = 38,748.93*x* + 3593.01	1.0000	0.026	0.079	18.39
6	275	0.16–10.00	*y* = 50,734.87*x* + 1491.47	1.0000	0.003	0.010	18.79
7	250	0.16–10.00	*y* = 39,806.34*x* − 3769.30	0.9980	0.009	0.027	23.96
8	250	1.56–100.00	*y* = 8363.50*x* + 2067.17	1.0000	0.071	0.216	36.18
9 ^b^	210	3.13–100.00	*y* = 9179.35*x* − 8.21	1.0000	0.764	2.315	9.27

^a^ Gallic acid (1), 5-(hydroxymethyl)furfural (2), morroniside (3), loganin (4), liquiritin apioside (5), liquiritin (6), ononin (7), glycyrrhizin (8), and allantoin (9). ^b^ Analysis of allantoin was conducted using a Luna NH_2_ column. ^c^ *r*^2^: coefficient of determination. ^d^ LOD: limit of detection. ^e^ LOQ: limit of quantification.

**Table 2:** Recovery (%) of the nine markers in the developed HPLC–PDA assay (n = 5).

Analyte	Original Amount (μg/mL)	Spiked Amount (μg/mL)	Detected Amount (μg/mL)	Recovery (%) ^a^	SD ^b^	RSD (%) ^c^
1	2.14	1.00	3.16	102.65	1.52	1.48
		2.00	4.17	101.79	1.17	1.15
		4.00	6.18	101.01	0.60	0.60
2	8.13	2.00	10.18	102.26	1.12	1.09
		4.00	12.21	101.95	1.01	0.99
		8.00	16.09	99.41	0.64	0.65
3	4.14	1.00	5.18	104.66	0.67	0.64
		2.00	6.07	96.63	0.43	0.45
		4.00	8.38	106.20	0.19	0.18
4	3.50	1.00	4.55	105.01	1.00	0.96
		2.00	5.57	103.50	0.29	0.28
		4.00	7.78	106.95	0.30	0.28
5	7.42	2.00	9.38	98.20	0.48	0.49
		4.00	11.68	106.49	0.63	0.59
		8.00	15.56	101.83	0.44	0.43
6	1.52	1.00	2.50	98.19	2.45	2.50
		2.00	3.56	102.21	0.58	0.57
		4.00	5.37	96.36	0.74	0.76
7	1.50	1.00	2.50	100.37	0.57	0.57
		2.00	3.50	99.73	0.21	0.21
		4.00	5.64	103.56	0.10	0.10
8	37.02	7.00	44.37	105.00	0.22	0.21
		17.50	54.57	100.28	0.21	0.21
		35.00	74.54	107.21	0.08	0.08
9	23.85	6.00	29.90	100.77	0.50	0.49
		15.00	39.62	105.10	1.39	1.32
		30.00	54.20	101.15	0.99	0.98

^a^ Recovery (%) = (original amount − detected amount)/spiked amount × 100. ^b^ SD: standard deviation. ^c^ RSD: relative standard deviation.

**Table 3:** Precision of the nine markers in the developed HPLC–PDA assay.

Analyte	Conc. (μg/mL)	Intraday (n = 5)			Interday (n = 15)		
		Observed Conc. (μg/mL)	Precision (RSD, %)	Accuracy (%)	Observed Conc. (μg/mL)	Precision (RSD, %)	Accuracy (%)
1	2.5	2.50	0.37	99.86	2.47	1.19	98.90
	5.0	4.95	0.67	99.04	4.93	0.66	98.70
	10.0	9.88	0.12	98.78	9.82	0.64	98.18
2	5.0	4.99	0.43	99.77	5.03	0.96	100.55
	10.0	10.02	0.72	100.15	10.07	0.75	100.72
	20.0	20.11	0.32	100.55	20.30	0.92	101.48
3	2.5	2.53	0.44	101.18	2.54	0.67	101.74
	5.0	5.04	0.34	100.85	5.09	1.08	101.87
	10.0	10.03	0.33	100.26	10.10	0.77	100.95
4	2.5	2.50	0.60	99.96	2.52	0.99	100.71
	5.0	5.00	0.41	100.04	5.11	1.01	102.21
	10.0	10.00	0.51	100.04	10.07	0.77	100.70
5	7.5	7.62	0.57	101.58	7.66	0.71	102.12
	15.0	15.14	0.50	100.91	15.30	1.11	101.98
	30.0	30.05	0.35	100.18	30.26	0.78	100.87
6	2.5	2.54	0.53	101.63	2.55	0.73	102.13
	5.0	5.05	0.58	100.94	5.10	1.12	102.01
	10.0	10.02	0.33	100.21	10.09	0.77	100.90
7	2.5	2.53	0.61	101.13	2.54	0.68	101.66
	5.0	5.01	0.38	100.24	5.07	1.11	101.32
	10.0	10.18	0.37	101.75	10.25	0.80	102.49
8	25.0	25.40	0.53	101.58	25.51	0.65	102.04
	50.0	50.45	0.43	100.90	50.93	0.99	101.86
	100.0	100.28	0.39	100.28	100.96	0.77	100.96
9	25.0	24.99	0.47	99.97	24.89	0.45	99.58
	50.0	49.88	0.26	99.75	49.99	0.63	99.98
	100.0	98.08	0.10	98.08	98.17	0.44	98.17

**Table 4:** Concentrations of nine marker compounds in JGE samples using HPLC–PDA assay (n = 3).

Analyte	JGE–1		JGE–2		JGE–3	
	Mean (mg/g) ± SD (×10^–2^)	RSD (%)	Mean (mg/g) ± SD (×10^–2^)	RSD (%)	Mean (mg/g) ± SD (×10^–2^)	RSD (%)
1	0.21 ± 0.10	0.47	0.21 ± 0.25	1.23	0.21 ± 0.24	1.16
2	0.81 ± 0.17	0.21	0.82 ± 0.08	0.10	0.82 ± 0.08	0.09
3	0.41 ± 0.02	0.04	0.41 ± 0.01	0.02	0.41 ± 0.03	0.06
4	0.35 ± 0.10	0.29	0.35 ± 0.01	0.04	0.35 ± 0.04	0.13
5	0.74 ± 0.24	0.32	0.74 ± 0.06	0.08	0.75 ± 0.14	0.19
6	0.15 ± 0.23	1.50	0.15 ± 0.04	0.30	0.15 ± 0.18	1.17
7	0.15 ± 0.10	0.69	0.15 ± 0.01	0.08	0.15 ± 0.08	0.52
8	3.69 ± 0.45	0.12	3.68 ± 0.17	0.05	3.67 ± 0.24	0.06
9	2.94 ± 0.62	0.21	2.92 ± 0.95	0.33	2.88 ± 0.16	0.06

## Data Availability

All data are available in this article and Supplementary Materials.

## Supplementary Materials

The following supporting information can be downloaded at: https://www.mdpi.com/article/10.3390/ph18040481/s1, Figure S1: Chemical structures of the nine analytes selected as marker compounds for quality control of Jwagwieum; Figure S2: HPLC profiling of six herbal medicines and their reported main constituents—A: *R. glutinosa*; B: *D. japonica*; C: *L. chinensis*; D: *C. officinalis*; E: *P. cocos*; F: *G. uralensis*; Figure S3: HPLC chromatograms of a standard solution containing 16 reference standards for testing (A) and 70% methanol solution of Jwagwieum water extract (B) monitored at various wavelengths—gallic acid (1), 5-(hydroxymethyl)furfural (2), protocatechuic acid (3), morroniside (4), methyl gallate (5), loganin (6), cornin (7), liquiritin apioside (8), liquiritin (9), cornuside (10), ononin (11), sweroside (12), liquiritigenin (13), glycyrrhizin (14), polyporenic acid C (15), and pachymic acid (16); Figure S4: Comparison of HPLC chromatograms of marker compounds according to various columns—A: SunFire^TM^, B: Kinetex, C: Capcell pak UG80, and D: Hypersil GOLD C_18_—gallic acid (1), 5-(hydroxymethyl)furfural (2), morroniside (3), loganin (4), liquiritin apoiside (5), liquiritin (6), ononin (7), and glycyrrhizin (8); Figure S5: Comparison of HPLC chromatograms of marker compounds according to various acids added to the mobile phase—A: 0.1% (*v*/*v*) formic acid, B: 0.1% (*v*/*v*) trifluoroacetic acid, C: 0.1% (*v*/*v*) phosphoric acid, and D: 1.0% (*v*/*v*) acetic acid—gallic acid (1), 5-(hydroxymethyl)furfural (2), morroniside (3), loganin (4), liquiritin apoiside (5), liquiritin (6), ononin (7), and glycyrrhizin (8); Figure S6: Comparison of HPLC chromatograms of marker compounds according to the temperature of the column oven—A: 30 °C, B: 40 °C, and C: 50 °C—gallic acid (1), 5-(hydroxymethyl)furfural (2), morroniside (3), loganin (4), liquiritin apoiside (5), liquiritin (6), ononin (7), and glycyrrhizin (8); Table S1: HPLC operating conditions for the simultaneous determination of Jwagwieum; Table S2: Equipment performance testing using retention time of nine marker analytes; Table S3: Equipment performance testing using peak area of nine marker analytes; Table S4: Stability test of nine marker compounds using the standard solution; Table S5: Stability test of nine marker compounds using the sample solution; Table S6: System suitability for the peak performance of the eight compounds; Table S7: Composition of Jwagwieum; Table S8: Information on nine reference standard compounds selected as marker analytes for quality assessment of Jwagwieum.
