# Supplementary material for: Development and Validation of an HPLC–PDA Method for Quality Control of Jwagwieum, an Herbal Medicine Prescription: Simultaneous Analysis of Nine Marker Compounds
Source: Pharmaceuticals (Basel). 2025 Mar 27;18(4):481. doi: 10.3390/ph18040481 (PMC12030021; doi:10.3390/ph18040481)
Supplement: Supplementary file 1 [file pharmaceuticals-18-00481-s001.zip › pharmaceuticals-3521046-supplementary.pdf]

Table S1.

HPLC operating conditions for the simultaneous determination of Jwagwieum.

**Parameters for the eight marker compounds**			
Column	SunFire™ C_{18} (250 mm × 4.6 mm, 5 μm)		
Detector	Photodiode array (monitoring at 235, 240, 250, 270, 275, and 280 nm)		
Flow rate (mL/min)	1.0		
Injection volume (μL)	10.0		
Column temperature (°C)	40.0		
Mobile phase	Solvent A: 0.1% (v/v) formic acid in distilled water<br>Solvent B: 0.1% (v/v) formic acid in acetonitrile		
Gradient flow	Time (min)	Solvent A (%)	Solvent B (%)
	0	95	5
	40	40	60
	50	5	95
	55	5	95
	60	95	5
	70	95	5
**Parameters for the allantoin**			
Column	Luna NH_2 (250 mm × 4.6 mm, 5 μm)		
Detector	Photodiode array (monitoring at 210 nm)		
Flow rate (mL/min)	1.0		
Injection volume (μL)	10.0		
Column temperature (°C)	40.0		
Mobile phase	Distilled water : Acetonitrile = 20 : 80		

Table S2

Equipment performance testing using retention time of nine marker analytes.

Analyte[a]	No. (#), retention time (min)						Mean	SD	RSD (%)
	1	2	3	4	5	6			
1	5.985	5.986	5.985	5.990	5.988	5.989	5.987	0.002	0.036
2	8.340	8.336	8.340	8.343	8.341	8.343	8.341	0.003	0.031
3	12.419	12.413	12.422	12.427	12.424	12.429	12.422	0.006	0.047
4	14.709	14.702	14.712	14.719	14.715	14.724	14.714	0.008	0.052
5	18.385	18.379	18.390	18.398	18.396	18.401	18.392	0.008	0.046
6	18.780	18.774	18.785	18.793	18.791	18.795	18.786	0.008	0.044
7	23.949	23.947	23.958	23.956	23.963	23.964	23.956	0.007	0.029
8	36.188	36.190	36.201	36.117	36.205	36.202	36.184	0.033	0.092
9	9.280	9.274	9.268	9.265	9.260	9.265	9.269	0.007	0.078

[a] Gallic acid (1), 5-(hydroxymethyl)furfural (2), morroniside (3), loganin (4), liquiritin apioside (5), liquiritin (6), ononin (7), glycyrrhizin (8), and allantoin (9).

Table S3

Equipment performance testing using peak area of nine marker analytes.

Analyte[a]	No. (#), peak area						Mean	SD	RSD (%)
	1	2	3	4	5	6			
1	1,203,520	1,210,726	1,210,296	1,197,841	1,197,393	1,197,057	1,202,805.50	6424.00	0.53
2	1,781,375	1,790,336	1,789,200	1,778,477	1,771,402	1,778,856	1,781,607.67	7147.17	0.40
3	1,142,083	1,149,810	1,148,595	1,142,267	1,139,312	1,142,014	1,144,013.50	4182.55	0.37
4	698,267	701,637	701,806	698,550	697,135	700,276	699,611.83	1919.77	0.27
5	865,392	870,486	870,445	864,646	862,427	864,812	866,368.00	3330.18	0.38
6	1,130,198	1,133,889	1,137,456	1,128,030	1,126,271	1,127,230	1,130,512.33	4350.63	0.39
7	770,842	776,137	776,241	772,165	769,755	770,642	772,630.33	2862.49	0.37
8	981,951	987,498	987,947	981,287	979,606	981,153	983,240.33	3558.75	0.36
9	908,125	910,278	909,390	906,486	906,621	904,912	907,635.33	2003.43	0.22

[a] Gallic acid (1), 5-(hydroxymethyl)furfural (2), morroniside (3), loganin (4), liquiritin apioside (5), liquiritin (6), ononin (7), glycyrrhizin (8), and allantoin (9).

Table S4

Stability test of nine marker compounds using the standard solution.

Analyte[a]	Time (h)							Mean (%)	SD	RSD (%)
	0	6	12	24	36	48	72			
1	100.00	99.97	99.82	98.90	98.40	99.87	104.70	100.24	2.06	2.06
2	100.00	100.36	100.33	100.82	101.69	102.93	105.24	101.63	1.89	1.86
3	100.00	100.40	100.53	100.61	101.03	103.04	104.40	101.43	1.64	1.62
4	100.00	101.25	100.84	101.53	101.05	103.24	104.96	101.84	1.69	1.66
5	100.00	100.58	100.64	100.73	101.13	103.18	104.57	101.55	1.67	1.65
6	100.00	100.68	100.78	100.88	101.07	103.19	104.65	101.61	1.67	1.64
7	100.00	100.62	100.71	100.77	101.11	103.29	104.66	101.59	1.71	1.68
8	100.00	100.75	100.80	100.84	101.17	103.21	104.70	101.64	1.68	1.65
9	100.00	100.14	100.67	99.86	99.91	101.08	103.26	100.70	1.21	1.21

[a] Gallic acid (1), 5-(hydroxymethyl)furfural (2), morroniside (3), loganin (4), liquiritin apioside (5), liquiritin (6), ononin (7), glycyrrhizin (8), and allantoin (9).

Table S5

Stability test of nine marker compounds using the sample solution.

Analyte[a]	Time (h)							Mean (%)	SD	RSD (%)
	0	6	12	24	36	48	72			
1	100.00	102.46	101.49	101.42	99.82	100.95	103.53	101.38	1.31	1.30
2	100.00	101.31	101.83	101.92	101.61	102.99	102.91	101.80	1.02	1.00
3	100.00	100.07	100.14	100.19	100.46	100.64	100.63	100.30	0.27	0.27
4	100.00	100.06	99.70	99.61	99.59	99.91	99.69	99.79	0.19	0.19
5	100.00	99.75	100.05	100.59	100.35	100.54	100.24	100.22	0.31	0.31
6	100.00	100.24	102.44	101.92	101.37	102.94	103.79	101.81	1.39	1.36
7	100.00	99.91	100.61	100.81	101.63	101.58	102.48	101.00	0.94	0.93
8	100.00	100.10	100.05	99.93	100.13	100.22	100.21	100.09	0.11	0.11
9	100.00	102.08	101.83	100.20	97.14	100.09	99.23	100.08	1.66	1.66

[a] Gallic acid (1), 5-(hydroxymethyl)furfural (2), morroniside (3), loganin (4), liquiritin apioside (5), liquiritin (6), ononin (7), glycyrrhizin (8), and allantoin (9).

Table S6
System suitability for the peak performance of the eight compounds.

Analyte[a]	Parameter[b]				
	k	α	*N*	*Rs*	*S*
1	1.16	—	16,566.18	8.40	0.99
2	2.00	1.73	52,931.73	8.40	1.08
3	3.47	1.73	139,505.06	12.63	0.98
4	4.30	1.24	527,613.91	12.63	1.11
5	5.62	1.31	472,367.16	2.25	1.16
6	5.76	1.03	564,301.44	2.25	1.10
7	7.62	1.32	917,687.36	30.50	1.08
8	12.03	1.58	1,220,974.39	62.52	1.19

[a] Gallic acid (1), 5-(hydroxymethyl)furfural (2), morroniside (3), loganin (4), liquiritin apioside (5), liquiritin (6), ononin (7), and glycyrrhizin (8).

[b] Parameters: *k*; retention factor, α; selectivity factor, *N*; theoretical plate number, *Rs*; resolution, and *S*; symmetry factor.

Table S7

Composition of Jwagwieum.

Herbal name	Scientific name	Family	Using part	Origin	Manufacturing No.	Amount (g)	Ratio (%)
Rehmanniae Radix Preparata	*Rehmannia glutinosa* (Gaertn.) DC.	Plantaginaceae	Root	China	SHG-001-001-23-021	900.00	28.57
Dioscoreae Rhizoma	*Dioscorea japonica* Thunb.	Dioscoreaceae	Rhizome	Yeongcheon, Korea	SQ-22032-1	600.00	19.05
Lycii Fructus	*Lycium chinense* Mill.	Solanaceae	Fruit	Cheongyang, Korea	SQ-22013-2	600.00	19.05
Corni Fructus	*Cornus officinalis* Siebold & Zucc.	Cornaceae	Fruit	Gurye, Korea	SQ-23031-1	450.00	14.29
Poria Sclerotium	*Porica cocos* Wolf	Polyporaceae	Sclerotium	Yeongwol, Korea	SQ-22029-1	300.00	9.52
Glycyrrhizae Radix et Rhizoma Preparata cum Melle	*Glycyrrhiza uralensis* Fisch.	Leguminosae	Root and rhizome	Uzbekistan	CK22-G146-2-533	300.00	9.52
					Total	3150.00	100.00

Table S8

Information on nine reference standard compounds selected as marker analytes for quality assessment of Jwagwieum.

Analyte[a]	Purity (%)	Molecular formula	Molecular weight (*m*/*z*)	CAS No.	PubChem CID	Catalog No.	Maker
1	100.0	$C_7H_6O_5$	170.12	149-91-7	370	G7384	Merck KGaA
2	99.5	$C_6H_6O_3$	126.11	67-47-0	237332	CFN97149	Wuhan ChemFaces Biochemical
3	99.9	$C_{17}H_{26}O_{11}$	406.38	25406-64-8	11228693	BP0960	Biopurify Phytochemicals
4	≥ 98.0	$C_{17}H_{26}O_{10}$	390.38	18524-94-2	87691	BP0884	Biopurify Phytochemicals
5	99.6	$C_{26}H_{30}O_{13}$	550.51	74639-14-8	10076238	DR10690	Shanghai Sunny Biotech
6	99.6	$C_{21}H_{22}O_9$	418.39	551-15-5	503737	BP0874	Biopurify Phytochemicals
7	98.5	$C_{22}H_{22}O_9$	430.41	486-62-4	442813	BP1031	Biopurify Phytochemicals
8	99.1	$C_{42}H_{62}O_{16}$	822.93	1405-86-3	14982	BP0682	Biopurify Phytochemicals
9	99.7	$C_4H_6N_4O_3$	158.12	97-59-6	204	05670	Merck KGaA

[a] Gallic acid (1), 5-(hydroxymethyl)furfural (2), morroniside (3), loganin (4), liquiritin apioside (5), liquiritin (6), ononin (7), glycyrrhizin (8), and allantoin (9).

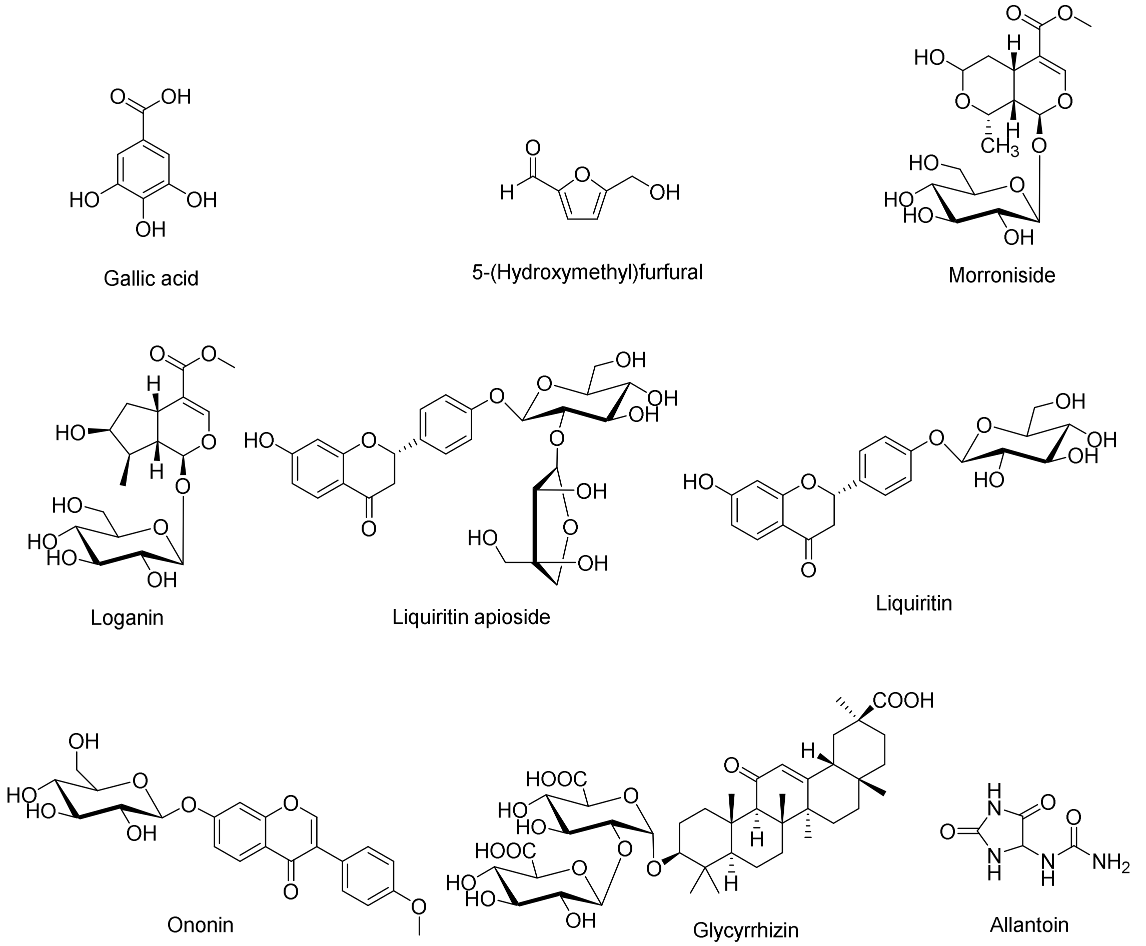

Figure S1. Chemical structures of the nine analytes selected as marker compounds for quality control of Jwagwieum.

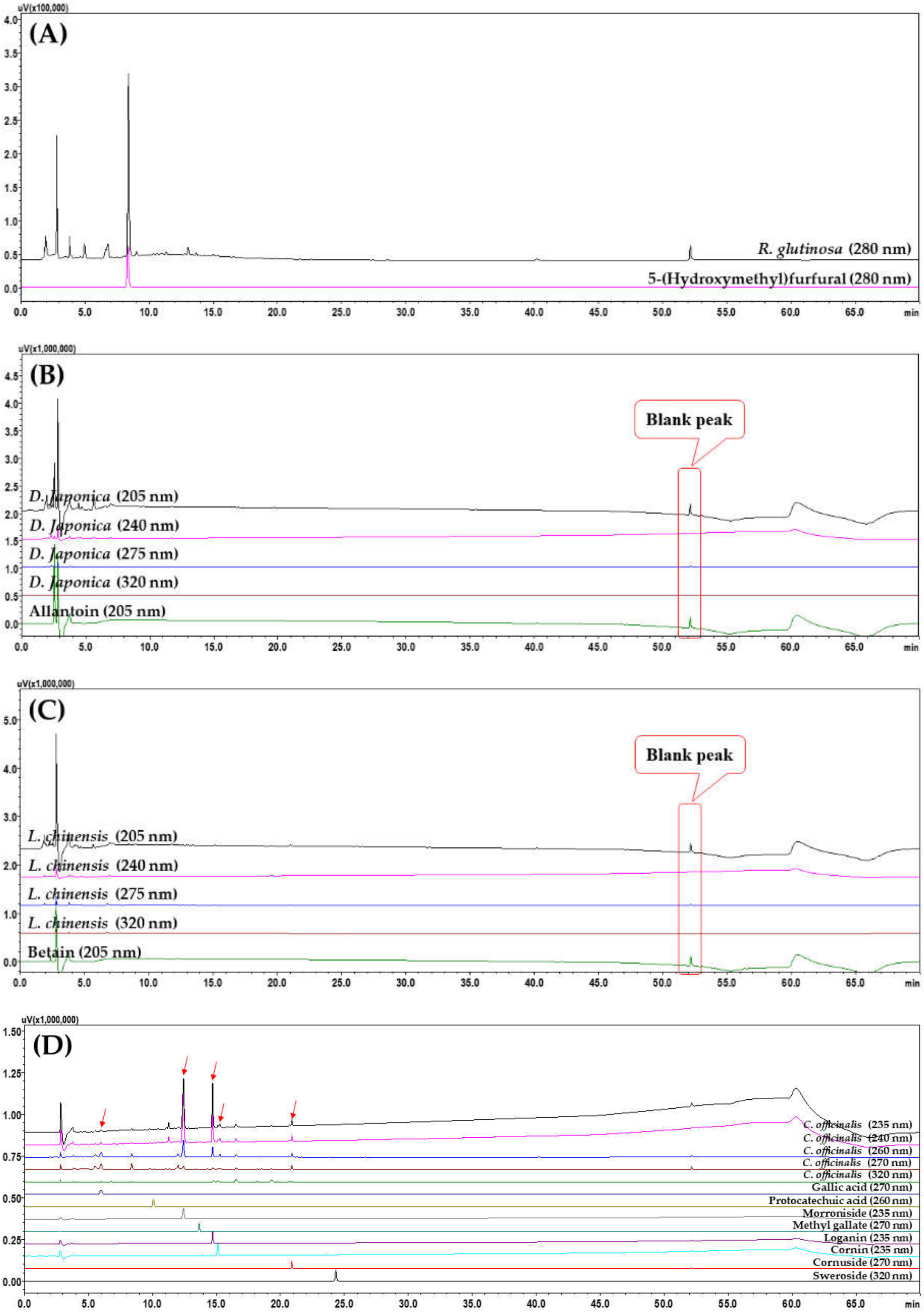
(A)
uV(x100,000)
R. glutinosa (280 nm)
5-(Hydroxymethyl)furfural (280 nm)
min
(B)
uV(x1,000,000)
Blank peak
D. Japonica (205 nm)
D. Japonica (240 nm)
D. Japonica (275 nm)
D. Japonica (320 nm)
Allantoin (205 nm)
min
(C)
uV(x1,000,000)
Blank peak
L. chinensis (205 nm)
L. chinensis (240 nm)
L. chinensis (275 nm)
L. chinensis (320 nm)
Betain (205 nm)
min
(D)
uV(x1,000,000)
C. officinalis (235 nm)
C. officinalis (240 nm)
C. officinalis (260 nm)
C. officinalis (270 nm)
C. officinalis (320 nm)
Gallic acid (270 nm)
Protocatechuic acid (260 nm)
Morroniside (235 nm)
Methyl gallate (270 nm)
Loganin (235 nm)
Cornin (235 nm)
Cornuside (270 nm)
Sweroside (320 nm)
min

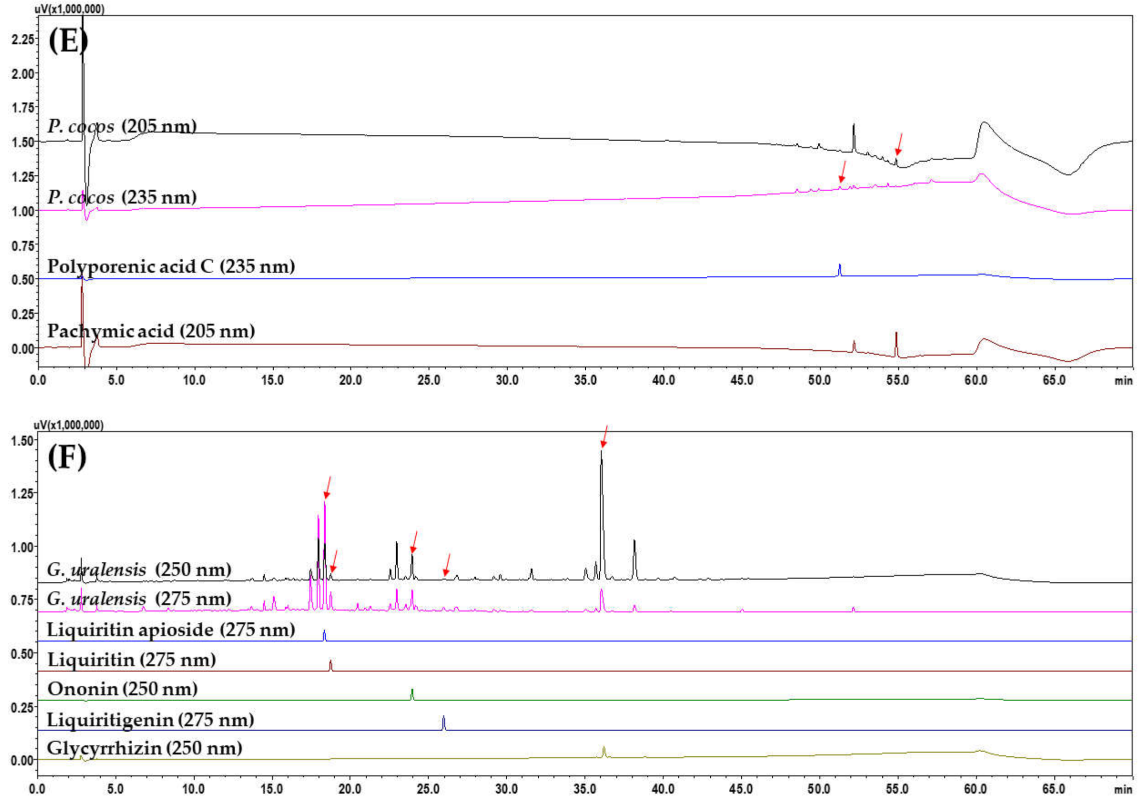

Figure S2. HPLC profiling of six herbal medicines and their reported main constituents—A: *R. glutinosa*; B: *D. japonica*; C: *L. chinensis*; D: *C. officinalis*; E: *P. cocos*; F: *G. uralensis*. Arrows indicate the peaks of marker compounds detected in each raw herbal medicine.

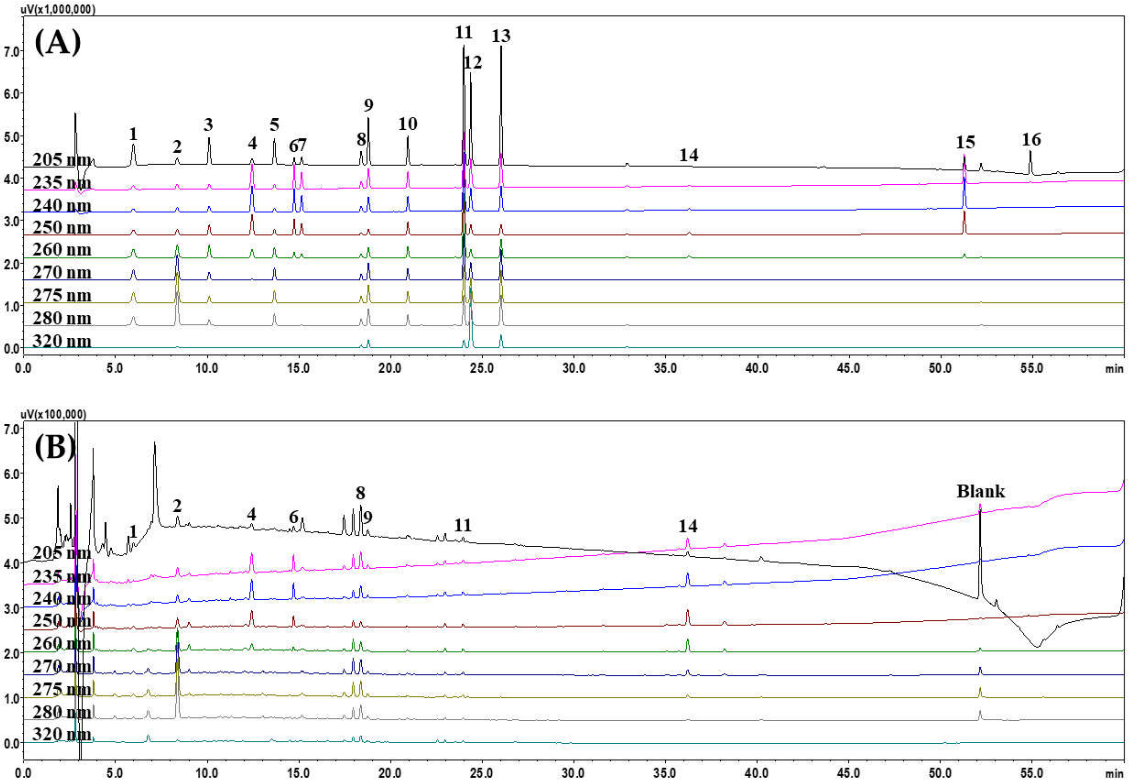

Figure S3. HPLC chromatograms of a standard solution containing 16 reference standards for testing (A) and 70% methanol solution of Jwagwieum water extract (B) monitored at various wavelengths—gallic acid (1), 5-(hydroxymethyl)furfural (2), protocatechuic acid (3), morroniside (4), methyl gallate (5), loganin (6), cornin (7), liquiritin apioside (8), liquiritin (9), cornuside (10), ononin (11), sweroside (12), liquiritigenin (13), glycyrrhizin (14), polyporenic acid C (15), and pachymic acid (16).

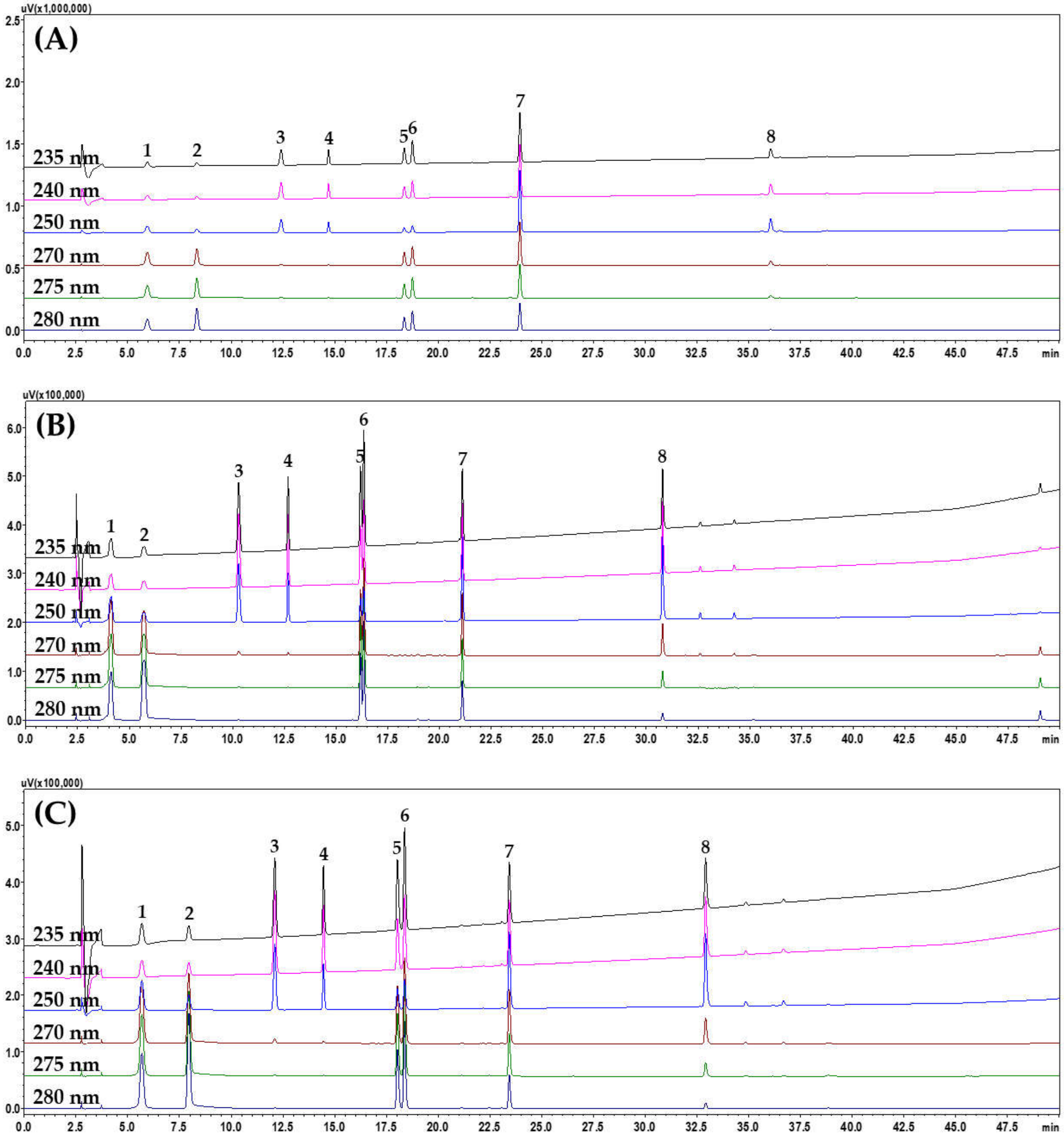
uV(x1,000,000)
(A)
uV(x100,000)
(B)
uV(x100,000)
(C)
235 nm
240 nm
250 nm
270 nm
275 nm
280 nm
1
2
3
4
5
6
7
8
min

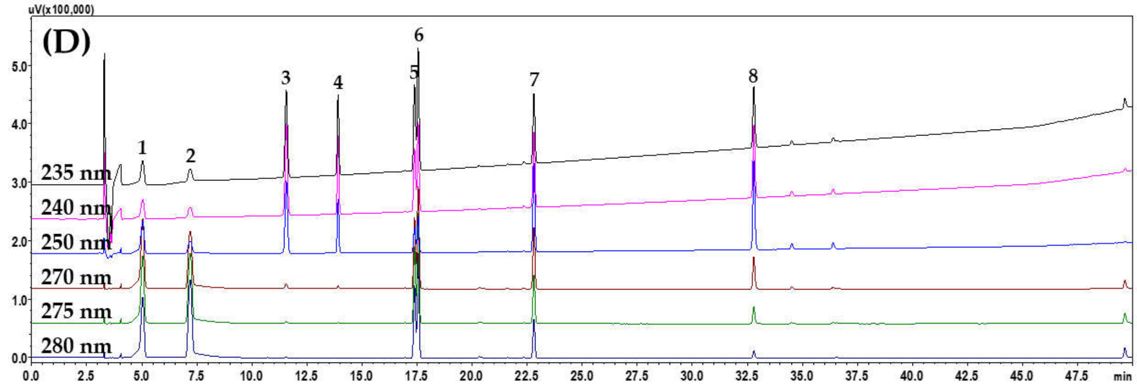

Figure S4. Comparison of HPLC chromatograms of marker compounds according to various columns—A; SunFire™, B; Kinetex, C; Capcell pak UG80, and D; Hypersil GOLD C_{18}—gallic acid (1), 5-(hydroxymethyl)furfural (2), morroniside (3), loganin (4), liquiritin apoiside (5), liquiritin (6), ononin (7), and glycyrrhizin (8).

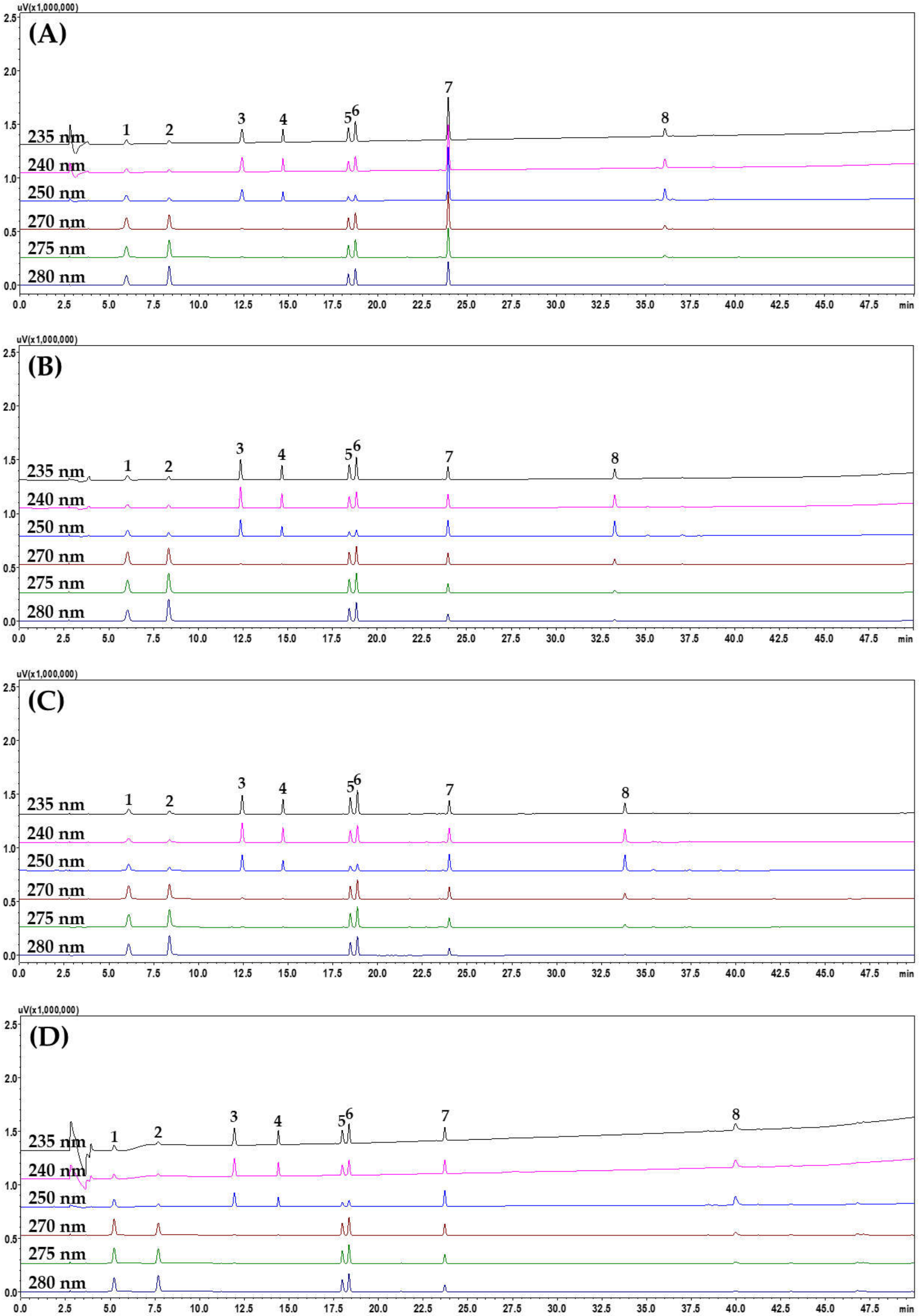

uV(x1,000,000)
(A)
(B)
(C)
(D)
235 nm
240 nm
250 nm
270 nm
275 nm
280 nm
1
2
3
4
5
6
7
8
min

Figure S5. Comparison of HPLC chromatograms of marker compounds according to various acids added to the mobile phase—A: 0.1% (v/v) formic acid, B: 0.1% (v/v) trifluoroacetic acid, C: 0.1% (v/v) phosphoric acid, and D: 1.0% (v/v) acetic acid—gallic acid (1), 5-(hydroxymethyl)furfural (2), morroniside (3), loganin (4), liquiritin apoiside (5), liquiritin (6), ononin (7), and glycyrrhizin (8).

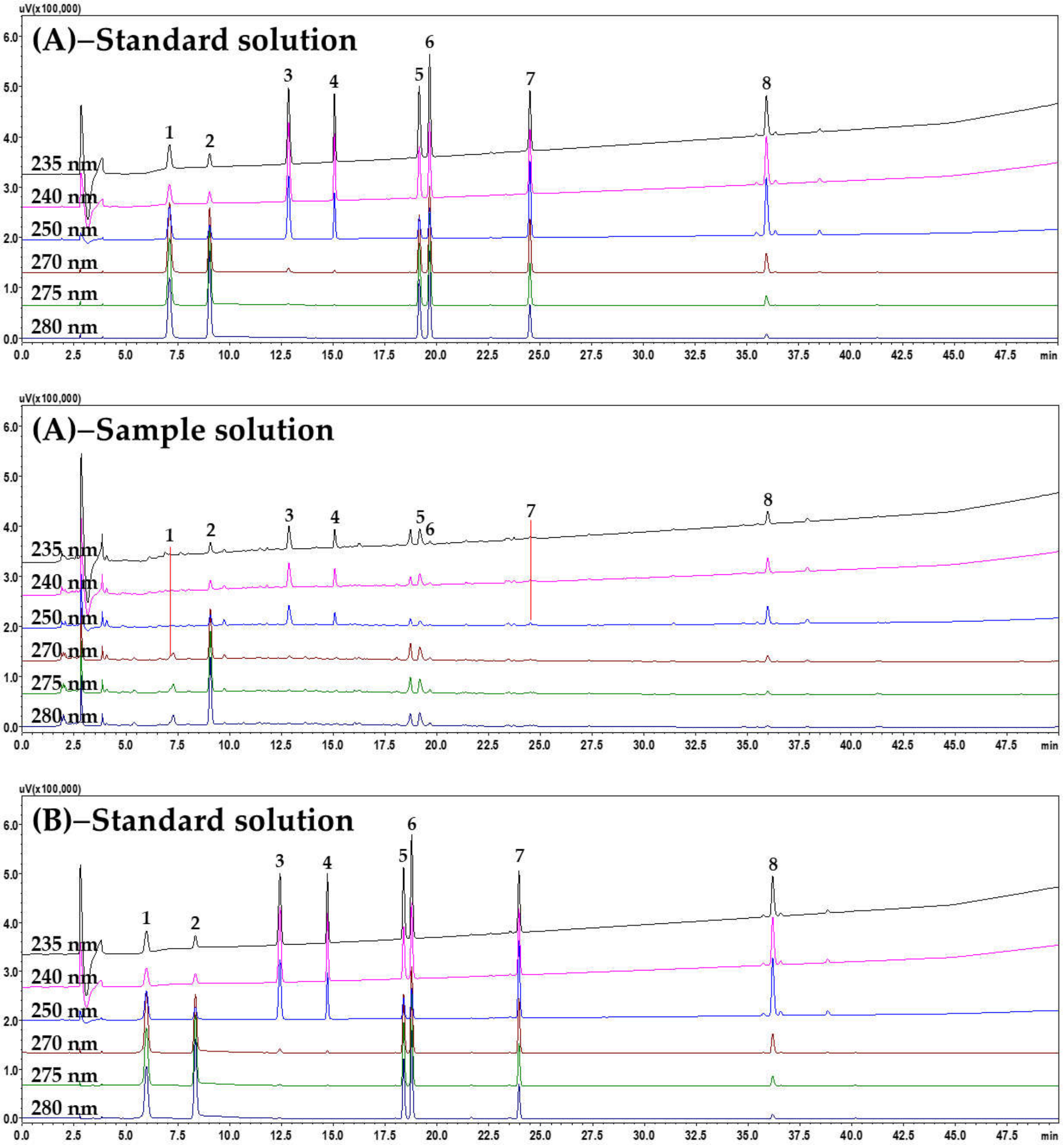
(A)–Standard solution
(A)–Sample solution
(B)–Standard solution
uV(x100,000)
235 nm
240 nm
250 nm
270 nm
275 nm
280 nm
min

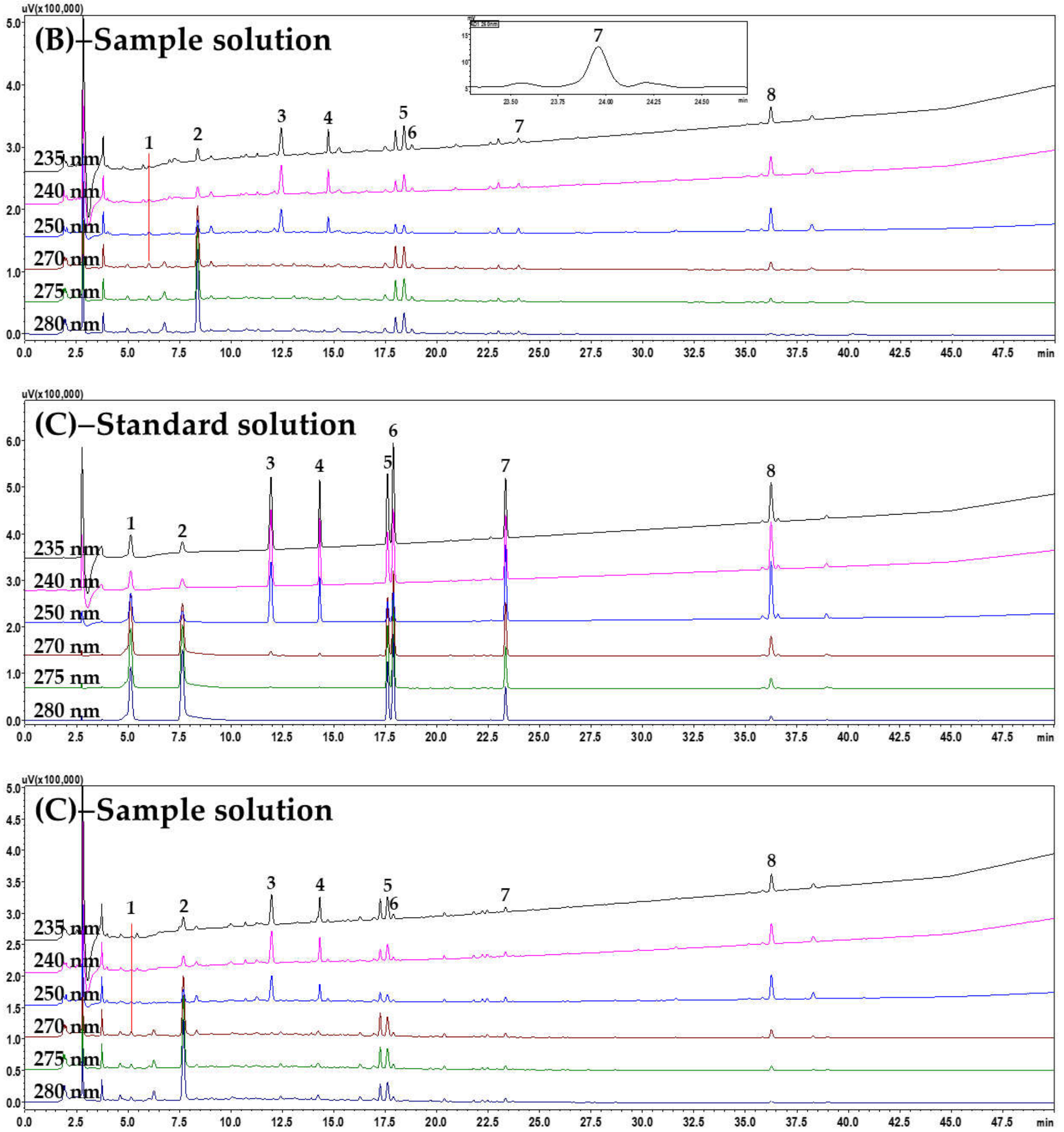

Figure S6. Comparison of HPLC chromatograms of marker compounds according to the temperature of the column oven—A: 30 °C, B: 40 °C, and C: 50 °C—gallic acid (1), 5-(hydroxymethyl)furfural (2), morroniside (3), loganin (4), liquiritin apoiside (5), liquiritin (6), ononin (7), and glycyrrhizin (8).
